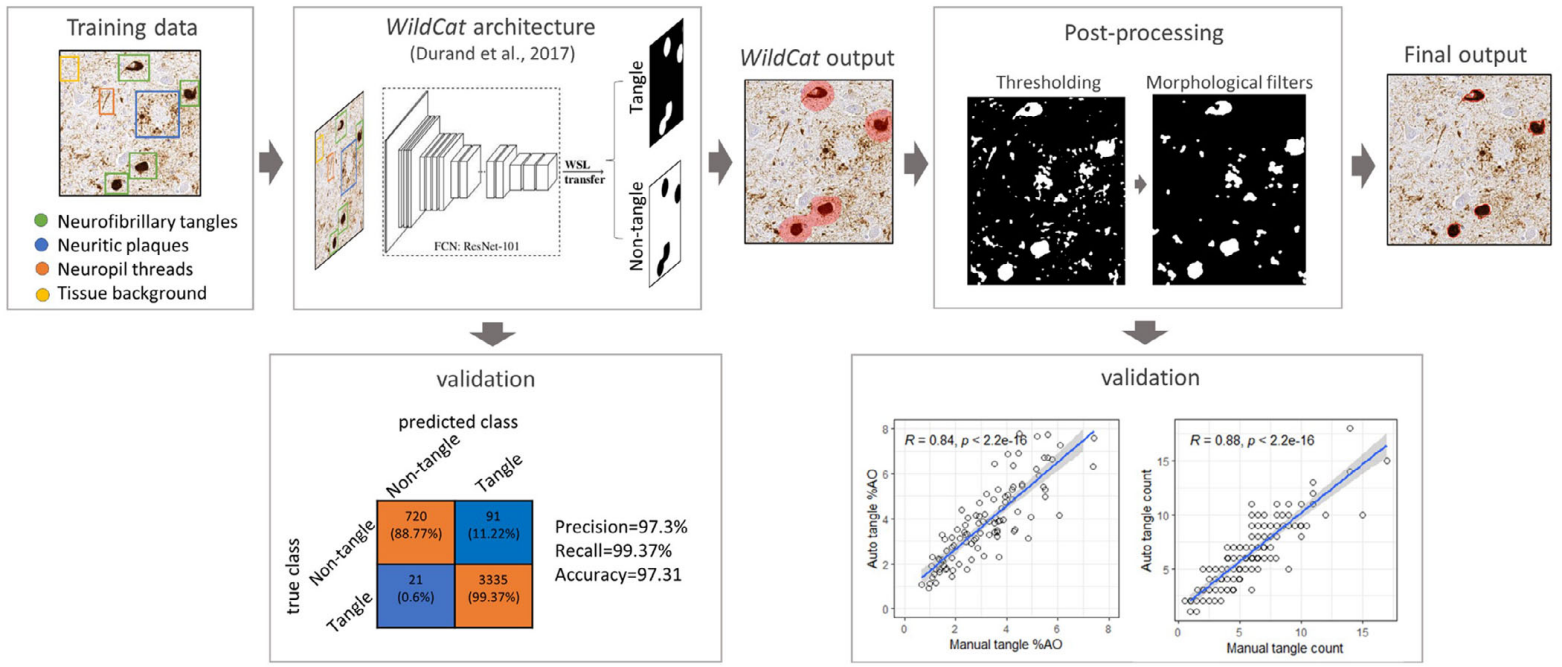# Automated Neurofibrillary tangle detections in digital histopathology sections of the brain

**DOI:** 10.1002/alz.092581

**Published:** 2025-01-03

**Authors:** Sanaz Arezoumandan, Eric Teunissen‐Bermeo, Noah Capp, Jamila Zablah, Daniel T Ohm, Jeffrey S Phillips, Corey T McMillan, Eddie B Lee, David A Wolk, Paul A. Yushkevich, David J Irwin

**Affiliations:** ^1^ University of Pennsylvania, Philadelphia, PA USA; ^2^ Department of Neurology, University of Pennsylvania, Philadelphia, PA USA; ^3^ Penn Frontotemporal Degeneration Center, Department of Neurology, Perelman School of Medicine, University of Pennsylvania, Philadelphia, PA USA

## Abstract

**Background:**

Alzheimer’s disease (AD) is the most common neurodegenerative disease, and it is characterized by aggregation of misfolded proteins in the brain. Intraneuronal accumulation of tau pathology form neurofibrillary tangles (NFT) in AD. The assessment of the severity of intraneuronal inclusions holds significance in studying the clinicopathological associations in neurodegenerative diseases. Using machine learning methods, we have developed an automated tool to detect NFTs in immunohistochemically stained sections.

**Method:**

We trained a weakly supervised machine learning algorithm called *WildCat* to classify inclusions in AT8‐stained 6µm sections of the hippocampus and locate areas with high probability of NFTs in the tissue. Next, we utilized the *WildCat* activation outputs and improved the NFT detections by thresholding and applying morphological filters. Finally, in order to validate our methods, we generated a validation dataset using *QuPath* software by manually annotating NFTs (N = 600) in 175 × 175µm regions of interest (N = 103). We then tested the correlation between manual and automated measurements to validate our method (**Fig. 1**).

**Result:**

The *WildCat* classification model showed outstanding performance by accurately distinguishing NFTs from non‐tangle inclusions, achieving high accuracy (97.31%), recall (99.37%), and precision (99.3%). In addition, we tested the correlation between final output of our model with manual measurements of tangles in our validation dataset, where we found strong correlations in tangle counts and tangle percent area‐occupied between the two methods (R = 0.84‐0.88, p<0.001) (**Fig. 1**).

**Conclusion:**

Automated tools can reliably generate measurements of NFT density in AD tissue sections. Moreover, this method can be applied to other types of pathologies and may offer a helpful tool for the study of other neurodegenerative diseases.